# Female high school students' knowledge and attitude toward breast cancer

**DOI:** 10.1186/s12905-023-02155-z

**Published:** 2023-01-30

**Authors:** Zahra Mohebi, Maryam Heidari Sarvestani, Zahra Moradi, Mohammad Mehdi Naghizadeh

**Affiliations:** 1 Student Research Committee, Fasa University of Medical Sciences, Fasa, Iran; 2 Sexual and Reproductive Health, School of Nursing and Midwifery, Isfahan University of Medical Science, Isfahan, Iran; 3 Department of Midwifery, School of Nursing, Fasa University of Medical Sciences, Fasa, Iran; 4 NonCommunicable Diseases Research Center, Fasa University of Medical Sciences, Fasa, Iran

**Keywords:** Students, Knowledge, Attitude, Breast self-examination, Breast cancer

## Abstract

**Background:**

Breast cancer is the most common cancer among women. Even though early detection and diagnosis of this disease can reduce mortality considerably, several studies have shown that more than 92% of women are unaware of the risk factors for breast cancer and of breast cancer screening tests. The simultaneous promotion of screening and provision of education can increase community health and reduce medical costs, and students can play a leading role in informing and educating people in society.

**Aim:**

The present study aims to examine the level of knowledge and attitude of female students in senior high schools in Fasa regarding breast cancer screening.

**Methods:**

In this cross-sectional study, the random sampling method was applied to survey 350 female high school students, only 311 of whom completed the study. Questionnaires were completed in girls' high schools, and the data was analyzed by SPSS Software, Version 25.

**Results:**

The results revealed that more than 87% of students were unaware or had incorrect information regarding breast cancer screening. The link between the type of breast cancer information sources and students' general knowledge was significant. Most students were aware of the risk of breast cancer, but they were uninformed of its symptoms, risk factors, and prevention strategies, and students with a family history of breast cancer had a higher score in terms of mammography knowledge than students with no family history of breast cancer, although this difference was not significant (mean ± standard deviation = 0.94 ± 0.90 vs. 0.67 ± 0.81, *p* = 0.19).

**Conclusion:**

The level of awareness and attitude of Fasa high school girls regarding breast cancer and screening methods is not acceptable. Therefore, it is recommended that educational programs be implemented to increase the awareness of students in schools, so that the number of screenings increases with the institutionalization of this information. Likewise, by transferring information through students to their families, the level of awareness in the whole society will hopefully increase as well.

## Background

After non-melanoma skin cancer, breast cancer is the second most common cancer in women aged 15–45 years, with an estimated 2.09 million new cancer cases and 626,679 deaths in 2018. It is also the most frequent malignancy among women in developing countries [1, 2]. Breast cancer (BC) is an important health issue all over the world, and based on the most recent Iranian National Population-based Cancer Registry survey, the age-specific incidence rate for breast cancer is about 34.53 per 100,000 [3]. Women aged 40–49 years form the group with the highest incident rate, and notably, women in Iran are affected by this disease 10 years earlier than women in other areas around the world. In 2017, it was estimated that 351.3 (275.2–374.2) disability-adjusted life years (DALY) were attributable to BC in Iran [4], where it is the fifth leading cause of cancer mortality. According to statistics, the probability of BC incidence for individuals aged 15–79 years in Iran has increased in the last 30 years [5]. This matter is dramatically worsened when we factor in Iran’s increasing cancer rate which was predicted to be 6.9% in 2020 and 115.7% in 2040 (compared to other countries: 5.0% in 2020 and 63.4% in 2040) [3]. Breast cancer in young women (< 40 years) is rare and carries a poor prognosis relative to BC in older women [6]. According to new statistics in Iran, 6160 breast malignancies are diagnosed in the country each year, and 1063 cases result in death. In addition, Iran has no national screening program for early diagnosis and control of BC, which is a significant factor in decreasing its burden. Furthermore, females’ awareness of BC warning signs and impressive screening is significantly insufficient [7].

The early detection and diagnosis of BC are ideal solutions for reducing mortality worldwide [8]. BC prevention based on awareness and early diagnosis are crucial in adolescents and young women because of the worse consequences they experience. It has been shown that adolescents and young women are more likely to be diagnosed with stage III/IV breast cancer and high-grade cancer cells than older adults [2]. The Global Burden of Disease (GBD) report announced an alarming increase in BC incidence, highlighting the importance of establishing procedures for the early detection and improved prognosis of this disease [9].

Breast cancer screening is conducted mainly through three methods: monthly breast self-examination, medical breast examinations, and breast imaging procedures under the direct supervision of a radiologist qualified in mammography [10]. Breast self-examination (BSE) is the most influential and feasible tool for the early detection of BC in developing countries [11].

In general, increasing women’s awareness of BC can positively affect their better performance of BSE. Awareness is the recollection of the details and the general procedures (i.e. recognition and review), processes, patterns, structures, or situations [12]. Adolescent girls are the main target population to receive education on breasts so as to develop positive habits, like BSE, because adolescence is a critical period for developing life habits and attitudes and forming health-related lifestyles, behaviors, habits, and attitudes in the future [13]. In developing countries, for young women to gain an adequate knowledge of BSE and to perform it, developing a consciousness of breast health and an awareness of BC at this age is vital [14].

The better health habits one has in adolescence, the better one’s health will be in adulthood. In other words, healthy habits, in particular BSE, may have profound and long-term effects on women’s health [15].

Proper health practices should be developed as early as possible and should lead to the lifetime maintenance of good health. Adolescent females are an important target group for the promotion of proper health habits, in particular with regards to breast health. Given that BC is a growing health problem and the knowledge of, attitude toward, and practice of BSE should be formed in adolescence. In author-conducted searches, no study was found that measured the knowledge and attitude of female high school students about BC in Iran. Therefore, the present study aims to examine the level of knowledge and attitude of female students in senior high schools in Fasa regarding BC screening.

## Methods

This analytic cross-sectional study was conducted in December, 2021, to evaluate female high school students’ knowledge of and attitude toward BC in Fasa. After obtaining approval for the proposed study from the Student Research Committee of Fasa University of Medical Sciences (research design code 99048; ethics code IR.FUMS.REC.1399.181) and the required permission from the Bureau of Education, Department of Safety and Security in Fasa, the researchers visited three female high schools in three different geographical areas of Fasa to cover a broad spectrum of students in terms of culture, education, and awareness. Then, the school principals allowed the participation of those students who provided informed consent before the study began. The inclusion criteria comprised female high school students and willingness to participate in the study. Questionnaires that were incomplete were excluded. All methods were carried out in accordance with relevant guidelines and regulations. All students were Persian speakers, and the questionnaire was designed in Persian language.

A two-section questionnaire on the students' level of knowledge and attitude regarding BC was distributed. The first section consisted of demographic information, including questions on students' age and field of study and on parents' age, education, and occupation. The second section consisted of questions about students' knowledge and attitudes about BC, the content validity of which was measured through surveys of ten gynecologists and faculty members of the nursing and midwifery department. The validity of the questionnaire was assessed using face and content validity methods. Quantitative face validity of the questionnaire was explored using impact scores. Impact scores > 1.5 represented the appropriateness of the items [16]. According to 20 midwifery instructors, the impact scores of all questionnaire items were higher than 1.5. To investigate content validity, the content validity ratio (CVR) and content validity index (CVI) were used. The necessity of the items was determined by the experts as “necessary,” “useful but not necessary,” and “not necessary” considering CVR. The opinions of ten midwifery instructors were collected, and values greater than 0.62 were considered acceptable based on the Lawshe table [17]. Experts were asked to evaluate and score the items in terms of relevance, clarity, and simplicity. Scores above 0.79 were considered acceptable. The total content validity of the questionnaire was computed using S-CVI/Ave where the minimum score of 0.79 was considered acceptable [18]. Based on the results, the S-CVI/Ave of the questionnaire was found to be 0.94, and its reliability was confirmed by a retest and Cronbach's alpha coefficient (Cronbach alpha r = 0.67).

The questionnaire comprised two sections that measured awareness and attitude. The first part consisted of 21 questions, including 6 on breast cancer awareness, 9 on knowledge of breast self-examination, 3 on knowledge of clinical examination, and 3 on knowledge of mammography. A correct answer was scored as 1, and a wrong answer or one answered “I don't know” was scored as 0; the sum of all questions was considered as the knowledge score. The second part comprised 15 five-point Likert-scale questions on attitude toward BC which were scored from 0 to 4 with values ranging from “strongly disagree” to “strongly agree.” The sum of all questions was considered as the attitude score. All scores were categorized according to their domain in three equal parts, namely "low," "moderate," and "good."

In Cochran’s sample size formula, the *p* = 0.5 gives a maximum sample size of 385 with d = 0.05 and confidence level = 0.95. This was modified in a population with N = 1500 female students, making the current sample size 306. To obtain a sufficient sample size, we selected 350 students, and ultimately, 311 qualifying questionnaires were analyzed. The cluster sampling method was applied to survey female high school students in different regions of Fasa city, located in the southeast of Fars province in Iran. Each class in a school was a cluster unit, and all of the students in the selected cluster were surveyed.

$$n = \frac{{2s^{2} }}{{d^{2} }}(Z_{1 - \alpha /2} + Z_{1 - \beta } + )^{2}$$

Descriptive statistics, including frequency rate, percentage, mean and standard deviation, are used to describe the characteristics of participants according to the findings. Awareness and attitude were also classified into three equal groups. Based on demographic information and a family history of breast cancer, the level of education and occupation of parents and the knowledge and attitude of students were compared using an independent t-test. The significance level was defined as 0.05, and the data was analyzed using SPSS version 25.

## Results

### Demographic information

The average ages of participants, students' mothers, and students' fathers were 17.46 ± 1.57, 47.42 ± 4.68, and 54.89 ± 6.89, respectively. The majority of parents had a high school diploma, with the prevalence being 83% and 74.65% for fathers and mothers, respectively. Fathers were mainly freelancers (69.54%), and mothers were housewives (83.42%). All students were studying either human sciences (64%) or mathematics and physics (36%). The students had gained their knowledge about BC mainly from books (27.3%); they had rarely referred to midwives or gynecologists to obtain information (8.4%). Only 5.4% of students had a family history of BC (Table 1).

**Table 1:** Frequency distribution of students' demographic characteristics

	N (%)	Mean ± SD
Do you have information about breast cancer?		
Yes	229 (73.6)	
No	82 (26.4)	
Source of information about breast cancer		
Book	85 (27.3)	
TV	64 (20.6)	
Doctor and midwife	26 (8.4)	
Friend	57 (18.3)	
Internet	33 (10.6)	
Don't know	46 (14.8)	
Family history of breast cancer		
Yes	17 (5.4)	
No	294 (94.5)	
Family relationship of the affected person with the student		
Mother	5 (1.6)	
Sister	2 (0.6)	
Aunt	10 (3.2)	
Negative	295 (0.94)	
Mothers' education		
Diploma	252 (81.02)	
Bachelor’s degree and higher	59 (18.98)	
Fathers' education		
Diploma	236 (75.88)	
Bachelor's degree and higher	75 (24.12)	
Student' Field of Study		
Mathematics and physics	112 (36.02)	
Human sciences	199 (63.98)	
Mothers' Job		
Housewives	259 (83.28)	
Employee	52 (16.72)	
Fathers' Job		
Freelancers	216 (69.45%)	
Employee	95 (30.55)	
Students' average ages		17.46 ± 1.57
Mothers' average ages		47.42 ± 4.68
Fathers' average ages		54.89 ± 6.89

### Knowledge of and attitude toward breast cancer and its screening methods

More than one third (35%) of the participating students thought that only women could suffer from BC. Overall, 41% of students were unaware of its symptoms, and 36.7% of students thought that painful lumps were a sign of BC. More than half of the students thought they would be at risk for the disease if they had a positive family history or were over 40 years old. More than 70% of them had no information (i.e., time and procedure) about BSE, nor had they received any education on the subject. Only 23% of students had been educated on BSE, and these had received information given mostly by midwives and gynecologists (10.3%). Most students were also unaware of other screening methods such as clinical examination and mammography (Table 2).

**Table 2:** Status of knowledge of high school female students about breast cancer and its screening methods (N = 311)

Variables	N (%)
*Breast Cancer Awareness*	
Do only women get BC?	
Yes	109 (35)
No	199 (64)
I don’t know	3 (1)
What are the symptoms of BC?	
Secretion of two breast	17 (5.5)
Touch the painful mass	114 (36.7)
Nipple troughs	34 (10.9)
I don’t know	128 (41.2)
Risk factors for BC	
Age > 40	211 (67.8)
Positive FH	57 (18.3)
Previous history of BC	13 (4.2)
Having children after the age of 30 or not having children	12 (3.9)
Premature menarche and late menopause	6 (1.9)
Obesity	12 (3.9)
Do you have information about BSE?	
Yes	93 (29.9)
No	218 (70.1)
Do you know BSE is a screening test?	
Yes	114 (36.7)
No	197 (63.3)
Have you had any training on how to do BSE?	
Yes	74 (23.8)
No	237 (76.2)
If so, by whom?	
Doctor or midwife	32 (10.3)
Parents	17 (5.5)
Friends	7 (2.3)
Teacher	7 (2.3)
Internet and cyberspace	26 (8.4)
No training	222 (71.4)
At what age should BSE begin?	
Correct answer	53 (17)
Wrong answer	258 (83)
How often should a BSE be performed?	
Correct answer	64 (22.06)
Wrong answer	247 (77.94)
When is the best time to do a BSE?	
Correct answer	69 (22.19)
Wrong answer	242 (77.81)
Who should do a BSE?	
Correct answer	80 (25.7)
Wrong answer	231 (74.3)
What are the stages of BSE?	
Correct answer	14 (4.5)
Wrong answer	277 (95.5)
What do you do if you find something abnormal during a BSE?	
Correct answer	237 (76.2)
Wrong answer	74 (23.8)
What are the benefits of BSE?	
Correct answer	143 (46.0)
Wrong answer	168 (54.0)
Who should do the clinical examination?	
Correct answer	269 (86.5)
Wrong answer	42 (13.5)
How is the clinical examination performed?	
Correct answer	28 (9.0)
Wrong answer	302 (91.0)
How often is a clinical breast examination performed?	
Correct answer	39 (12.5)
Wrong answer	272 (87.5)
Is mammography useful for early detection of breast cancer?	
Correct answer	124 (39.9)
Wrong answer	187 (60.1)
At what age should mammography be started?	
Correct answer	48 (15.4)
Wrong answer	263 (84.6)
How often should a mammogram be performed?	
Correct answer	43 (13.8)
Wrong answerr	268 (86.2)

*FH* Family History, *BC* breast cancer, *BSE* Breast self-examination

Students' attitudes about BC screening are shown in detail. Most of the students in the attitude survey had no opinion about the questions asked, and people who completely agreed or completely disagreed had the lowest number and percentage (Table 3).

**Table 3:** Attitudes of female high school students about breast cancer (N = 311)

Variables	Completely agree	Agree on	No idea	Opposed	Completely opposed
	N (%)	N (%)	N (%)	N (%)	N (%)
I'm too young to get breast cancer	80 (25.7)	33 (10.6)	78 (25.1)	65 (20.9)	55 (17.7)
I am so healthy that my body is resistant to breast cancer so I do not feel the need for a monthly self-examination	26 (8.4)	21 (6.8)	98 (31.5)	69 (22.2)	97 (31.2)
No smoking so no need to monthly BSE	14 (4.5)	13 (4.2)	97 (31.2)	96 (30.9)	91 (29.3)
No obesity so no need to monthly BSE	5 (1.6)	14 (4.5)	91 (29.3)	106 (34.1)	95 (30.5)
No eating fatty foods so no need to monthly BSE	9 (2.9)	16 (5.1)	99 (31.8)	110 (35.4)	77 (24.8)
No FH of BC so no need to practice BSE	8 (2.6)	37 (11.2)	102 (32.8)	107 (34.4)	57 (18.3)
Not having a stressful life so no need to practice BSE	9 (2.9)	32 (10.3)	104 (33.4)	108 (34.7)	58 (18.6)
If I feel pain in my breasts, the fear of breast cancer will take over my whole being	25 (8)	59 (19)	128 (41.2)	67 (21.5)	32 (10.3)
I think breast cancer is a serious disease because it eventually causes death	45 (14.5)	59 (19)	121 (38.9)	58 (18.6)	28 (9)
If I get breast cancer, I may not be able to get pregnant	14 (4.5)	41 (13.2)	183 (58.8)	43 (13.8)	30 (9.6)
I think breast cancer is more dangerous than other cancers	17 (5.5)	27 (8.7)	161 (51.8)	69 (22.2)	37 (11.9)
With timely treatment of breast cancer, a person will have a normal life	70 (22.5)	93 (29.9)	103 (33.1)	34 (10.9)	11 (3.5)
If I touch a painless lump in my breast, I'm afraid of breast cancer	23 (7.4)	93 (29.9)	131 (42.1)	43 (13.8)	21 (6.8)
In my opinion, breast cancer is dangerous and in addition to breast, it spreads the disease to other parts of the body	19 (6.1)	78 (25.1)	161 (51.8)	34 (10.9)	19 (6.1)
If my mother or sister gets breast cancer, I am at greater risk for breast cancer	45 (14.5)	87 (28.0)	143 (46)	14 (4.5)	22 (7.1)

The overall knowledge score was 7.15 ± 3.25, the highest mean score of which belonged to BSE (2.74 ± 1.68), and the lowest mean score belonged to mammography (0.69 ± 0.82). In addition, the overall score of attitudes was 35.45 ± 6.1. There was no relationship between students' level of knowledge and attitudes (*p* = 0.06) (Table 4).

**Table 4:** Average score of students' knowledge and attitude about breast cancer and its screening method variable

	Knowledge and attitude Mean ± SD	p
Breast cancer knowledge	2.64 ± 1.59	0.06
Knowledge ofBSE	2.74 ± 1.68	
Knowledge of clinical breast examination	1.08 ± 0.55	
Knowledge of mammography	0.69 ± 0.82	
General knowledge score	7.15 ± 3.25	
Attitude	35.45 ± 6.10	

Most of the participating students had an average attitude towards BC (73.31%). Most students had a weak general knowledge about BC (63.98%) (Table 5).

**Table 5:** Distribution of students based on knowledge score and attitude about breast cancer

Variable	Low	Moderate	Good
	N (%)	N (%)	N (%)
Breast cancer knowledge-cut	192 (61.73)	106 (34.08)	13 (4.11)
Knowledge of breast self-examination-cut	157 (50.48)	112 (36.01)	42 (13.5)
Knowledge of clinical breast examination-cut	53 (17.04)	255 (81.99)	3 (0.96)
Knowledge of mammography	171 (54.98)	134 (43.08)	6 (1.92)
General knowledge score-cut	199 (63.98)	103 (33.11)	9 (2.89)
Attitude-cut	23 (7.39)	228 (73.31)	60 (19.29)

There was no significant difference between the knowledge (*p* = 0.61) and attitude (*p* = 0.69) scores of students with and those without a family history of BC (Table 6). There was no significant difference between students’ attitudes and knowledge regarding their parents’ education and occupation (Table 7).

**Table 6:** Comparison of mean knowledge score and attitude of students in the two groups with and without family history of breast cancer

Variable	FH of BC		P-value
	Yes (n = 17)	No (n = 294)	
	Mean ± SD	Mean ± SD	
Breast cancer knowledge	2.29 ± 1.31	2.66 ± 1.60	0.35
Knowledge of breast self-examination	2.53 ± 1.37	2.75 ± 1.70	0.59
Knowledge of clinical breast examination	1.00 ± 0.61	1.08 ± 0.55	0.56
Knowledge of mammography	0.94 ± 0.90	0.67 ± 0.81	0.19
General knowledge score	6.76 ± 2.93	7.17 ± 3.28	0.61
Attitude	34.88 ± 6.48	35.48 ± 6.09	0.69

**Table 7:** Comparison of average knowledge score and students 'attitudes based on parents' level of education and occupation (N = 311)

Variable	General knowledge score	p	Attitude	p
	Mean ± SD		Mean ± SD	
*Father's education level*				
Diploma	6.76 ± 2.93	0.31	34.88 ± 6.48	0.36
Bachelor's degree and higher	6.89 ± 2.56		33.96 ± 5.99	
*Mother's education level*				
Diploma	6.78 ± 2.45	0.18	34.56 ± 6.23	0.29
Bachelor's degree and higher	7.03 ± 3.05		35.48 ± 6.09	
*Father's job*				
self-employment	6.85 ± 2.69	0.69	33.59 ± 6.03	0.56
Employee	6.59 ± 2.59		33.87 ± 6.12	
*Mother's job*				
Housewife	6.94 ± 2.62	0.65	34.01 ± 6.14	0.49
Employee	6.87 ± 2.49		33.74 ± 5.91	

## Discussion

In this study, the mean overall knowledge score of all students was low. Students had inadequate knowledge about BC, BSE, and mammography; however, they had moderate knowledge about clinical examination. The present results are consistent with those of Isara (2011), who evaluated the knowledge of 287 students in Nigeria and showed that most students had inadequate knowledge about BC (56.8%) and BSE (75.6%) [19]. Ogunkayode et al. (2021) studied the knowledge, attitude, and practice of BSE among 348 female secondary students aged 10 to 19 in Ibadan, Nigeria. They found that only 9.5% of high school students had good knowledge about BC and BSE [20]. Ibitoye (2019) studied 280 adolescent girls at the Fiwasaye girls' grammar school in Akure, Nigeria, and showed that students had relatively good knowledge about BC (60%) [1].

Mpulumba examined the knowledge of 962 students regarding BC and BSE in the Democratic Republic of the Congo. They discovered that most female students were aware of BC (61.75%) [21]. The results of the two studies mentioned above were not consistent with those of the current study, which may be explained by the poor education about BC in Iran.

Paknejad (2019) examined 2500 over-20 females in Tehran to measure their knowledge, attitude, and practice of self-examination and determined that 40.57% of women were aware of the signs and symptoms of BC, 23.03% of women were fairly aware of BSE, while the remaining women in the study had low and moderate knowledge [12]. Surprisingly, even though this study was conducted on married women over the age of 20 years, the participants were unaware of BC, which may be due to poor education about this disease rooted in cultural issues.

Karayurt et al. studied the awareness of BC risk factors and BSE of 718 students in Turkey and found that more than half of the students had misconceptions about BC and knew nothing about breast health. Moreover, one half or two thirds of the students knew little about breast health. A lack of education about BC has resulted in students having little knowledge of it, and thus, their awareness of it must be increased through proper education [15].

Many studies have shown that the level of cancer awareness is a significant risk factor for the early detection of BC and, in turn, vital for patient survival. Therefore, there is an urgent need for interventions to increase knowledge and awareness about BC and screening methods [22].

Most of the students in the present study (70.1%) had no information about BSE or had received no education about it. Those students who were informed about BSE had gained their information first from gynecologists and midwives and then via the Internet. Only 36% of students knew BSE was a screening test. Most of them were unaware of the suitable age, intervals, best time, and steps to start the BSE. Most of them knew that they had to refer to doctors or midwives for clinical examinations, but they were unaware of the methods and the intervals. Most of the students did not know mammography is used to look for BC and were unaware of the methods and the age at which to start looking for BC.

Mpulumba showed that most students were unaware of BSE methods, and the results were similar to those of the present study [21].

Similar to the present study, Ranasinghe showed that only 17.1% of study participants were aware of the methods of BSE, and only 9.4% knew BC screening is available. Only 35.6% of students knew mammography is an effective screening method [23].

Ogunkayode et al. showed that most students (74.6%) did not know any BSE methods. Less than half (31.9%) of the study participants were aware of the necessity for monthly BSE, and only 15.2% were aware of carrying out this examination even after menstruation. Moreover, 74.6% of students mentioned a lack of awareness as the main reason for not performing BSE [20].

Sadoh indicated that although 70% of students had heard of BSE, only 60% knew how to perform BSE to detect BC, and few students knew about mammography. Most students had no formal knowledge of examinations. They were unaware of some of the essential steps of BSE, such as examination of the axillary lymph nodes while holding both hands above the head. They did not even know that the best position for the palpation of the breast is lying down [2].

Karayurt showed that female high school students did not have adequate information about BSE, and only a small percentage of students performed monthly breast self-examinations. Most students did not perform BSEs because of a lack of knowledge of the methods (98.5%). This result was confirmed in a study of adolescent girls in Turkey. Only a few of them were aware of the best time for self-examination (13.2%), the frequency of self-examination (21.8%), and the correct methods of self-examination (26.6%) [15].

In Ghana, Fondjo compared 359 female secondary school students aged 15–19 to 677 tertiary school students aged 20–24 and found that almost all of the students were aware of BSE, indicating a significant difference between the level of knowledge of the two groups (33.0% and 67.0%, respectively). Moreover, 91.6% of students knew that BSE is a tool for diagnosing BC [11].

Many people think that adolescents are immune to cancer, and this false belief may make adolescents, like everyone else, reluctant to accept that they are vulnerable to this disease [12]. Therefore, raising awareness can improve future health-seeking behaviors (HSB), such as the application of screening tools. Although BSE has not reduced breast cancer mortality, the WHO recommends that high-risk individuals should perform it [2]. It is emphasized in every country, even in developed countries with organized mammography programs, that women should be aware of normal breasts and report any changes in their breasts to a healthcare provider immediately, because it has been proven that breast cancers are mainly self-diagnosed. One study conducted on black women found that BSE is four to five times as effective as mammography in BC diagnoses, which highlights the necessity of BSE by women [2], as 90% of breast masses are detected by women themselves [21].

Most BC patients have no family history of the disease. Therefore, it is, in fact, essential to provide students with better education about the benefits of BSE for early detection and diagnosis [11]. Recent studies have indicated that most women do not have correct information about BC risk factors and signs and symptoms [22].

The present study has shown that most of the participants knew BC is not just a woman’s disease; this result is inconsistent with Sadoh’s study, in which most participants believed that it is just a woman’s disease [2].

The present study has also shown that most of the students (41.2%) were unaware of the symptoms of BC; however, they knew a painful breast lump was a sign of it. They also believed that old age and family history are risk factors for the disease, which is consistent with Sadoh's study, in which many adolescents were unfamiliar with the symptoms of BC [2].

Sadoh showed that students were unaware that breast lumps without weight loss could cause cancer or that bloody nipple discharge is not always a sign of cancer [2]. Karayurt's study showed that most students had little knowledge of the risk factors for BC, which is consistent with the present study. Students mainly identified the risk factors for BC as a personal history of the disease (68.7%) and a family history of the disease (67.0%). In other words, students were aware that BC is related to genetic factors, and awareness of BC, mainly because of the extensive coverage of this risk factor in Turkish media, has a positive effect on breast health among young women. It is believed that lifestyle changes may modify the risks of developing BC. In other words, lifestyle changes can affect young women and help them avoid risk factors [15].

Ranasinghe showed that out of 859 adolescents, almost 60% of them identified risk factors for BC as breast lump history, family history of BC, and radiation exposure. Although most of them knew that breast lumps are warning signs, they knew very little about other warning signs [23].

Mpulumba showed that 59.56% of girls did not know the risk factors for BC. Girls aged 19–22 had more information about this disease than younger girls (less than 14 years), indicating a statistically significant difference between them (*p* < 0.001) [21].

It is essential to raise female students' knowledge so as to enable them to develop sustainable attitudes and change their lifestyles through education about the risk factors for BC [21]. Many women do not have a health-related attitude toward the disease, and many of them, in particular women from developing countries, do not participate in screening programs [22].

The students participating in the present study gained their knowledge about BC mainly from books (27.3%) and television (20%); however, they rarely referred to midwives or gynecologists for information (8.4%). The results showed that organized training programs were not enough to increase breast health awareness. After books, the present study found that TV was the most critical source for information about BC. Ogunkayode et al. showed that students gain their knowledge about BC mainly from TV/radio (82.5%) and healthcare givers (81.6%) [13]. Karayurt indicated that nearly half of students had gained knowledge about BC and BSE from the media [15]. Ranasinghe also showed that students had gained knowledge about BC from television and newspapers [23].

These findings show that the media is one of the most important sources of information about BC and BSE, and the role of the media in collaborating with healthcare educators to transfer correct information about BC and BSE to adolescents is highlighted.

Given the importance of the issue at hand, it is vital to improve the education of female students about BC; however, school teachers may not be the best option for the following reasons: (a) they may be unfamiliar with the subject, and (b) they may not conduct the education due to cultural sensitivity. A study conducted in Iran revealed that school teachers are aware of the risk of BC, but only a very small percentage (6%) conduct breast self-examinations. Therefore, midwives and healthcare providers are the best options to educate students [24].

Mpulumba showed that most students (26.72%) gain information about BC through medical staff [21].

Health training programs should start in the early stages of life and can effectively change risk factors for BC. Therefore, healthcare providers can play an essential role in educating students and increasing their awareness of risk factors for BC and their impact on student behavior [15].

The current study showed that most students have a moderate attitude towards BC (73.31%); only 19% of the current participants had a good attitude towards BC. Again, this is due to the lack of student awareness.

In Paknejad’s study, 47.86% of students had a positive attitude about BSE [12]. Fondjo (2018) indicated that 97.1% of students had a good attitude toward BSE, and 96.3% of students confirmed its necessity [11]. Ogunkayode et al. showed that more than half of the students (56%) had a positive attitude towards BSE [20].

Although the current study showed no significant relationship between students' level of knowledge and attitudes (*p* = 0.06), other studies have shown that people with good knowledge are more likely to have a positive attitude towards BSE. Ogunkayode realized a significant positive relationship between attitude and knowledge about BC and BSE [20].

No significant difference was found in the current study between the knowledge score (*p* = 0.61) and attitude (*p* = 0.69) and parents' level of education and occupation of students with and without a family history of BC. Most of the students were also unaware that their lifestyle, such as lack of physical activity, obesity, and excessive alcohol consumption, increases BC risk [2].

Some studies have shown that many factors such as age, household income, and family history of cancer are related to women's level of knowledge and awareness about BC [22]. Karayurt showed no significant relationship between BSE, perceived income level, and family history of BC [15]. This difference between studies can be attributed to the low level of public awareness and the lack of adequate education.

It is essential for adolescents to adopt sensitive basic behavioral patterns about BSE and have sufficient deep information to ensure proper and effective self-examination in the present and future, because being unaware of something makes doing it impossible [20].

Adequate awareness of BC and BSE can affect attitudes towards self-examination and its performance, which can help prevent poor BSE performance and late detection of BC [20].

### Limitation

One of the limitations of the current study is a lack of agreement with the questionnaire; there is no comprehensive and useful questionnaire to measure knowledge and attitude about breast cancer. Even though the researchers visited three all-female high schools in three different geographical areas of Fasa to cover a broad spectrum of students in terms of levels of culture, education, and awareness, only students within a limited age range were investigated, and this may limit the possibility of generalizing the results to other communities of women.

## Conclusion

Health behaviors that develop during adolescence can improve future health and have lifelong effects. There is a need to increase teenage girls’ knowledge of BC and the benefits of early diagnosis. Caregivers should develop effective training programs to raise awareness about BC, its risk factors, and ways to prevent and diagnose it in teenage girls so as to aid them in developing good health habits from a young age. Effective BSE training through mass media is a challenge for cultural reasons, so health education in schools is a crucial strategy to ensure that all women are aware of this important act. Teenage female students can play an essential role in promoting early diagnosis methods for BC, especially BSE, as they can pass their knowledge on to friends and older women in their families. The more educated they become, the more aware women will be in society, a society that enables the transfer of sustainable knowledge. As a first step, periodical and continuous training of health system employees such as midwives should be implemented in schools. Likewise, instruction for girls' high school teachers should be implemented as continuous or in-service training, so that teachers can transfer such training to students. Finally, employing midwives in schools as health educators can greatly help increase the level of literacy regarding BC.

## Data Availability

The datasets used and/or analyzed in the current study are available from the corresponding author on reasonable request.

## Abbreviations

BSE: Breast self-examination
GBD: Global Burden of Disease
BC: Breast cancer
